# Highly Efficient Perovskite Solar Cells with Substantial Reduction of Lead Content

**DOI:** 10.1038/srep35705

**Published:** 2016-10-18

**Authors:** Chong Liu, Jiandong Fan, Hongliang Li, Cuiling Zhang, Yaohua Mai

**Affiliations:** 1 Institute of Photovoltaics, College of Physics Science and Technology, Hebei University, Baoding, 071002, China; 2 Institute of New Energy Technology, College of Information and Technology, Jinan University, Guangzhou, 510632, China

## Abstract

Despite organometal halide perovskite solar cells have recently exhibited a significant leap in efficiency, the Sn-based perovskite solar cells still suffer from low efficiency. Here, a series homogeneous CH_3_NH_3_Pb_(1−x)_Sn_x_I_3_ (0 ≤ x ≤ 1) perovskite thin films with full coverage were obtained via solvent engineering. In particular, the intermediate complexes of PbI_2_/(SnI_2_)∙(DMSO)_x_ were proved to retard the crystallization of CH_3_NH_3_SnI_3_, thus allowing the realization of high quality Sn-introduced perovskite thin films. The external quantum efficiency (EQE) of as-prepared solar cells were demonstrated to extend a broad absorption minimum over 50% in the wavelength range from 350 to 950 nm accompanied by a noteworthy absorption onset up to 1050 nm. The CH_3_NH_3_Pb_0.75_Sn_0.25_I_3_ perovskite solar cells with inverted structure were consequently realized with maximum power conversion efficiency (PCE) of 14.12%.

Perovskite solar cells have transfixed researchers in the solar energy field since its power conversion efficiency (PCE) was increased up to 22.1% in only 6 years, which outperformed other photovoltaic technologies^1^,^2^,^3^,^4^,^5^,^6^,^7^. Although soaring efficiencies enable this technology to be potentially commercialized, there remains skepticism concerning its intrinsic toxicity and instability^8^,^9^,^10^. The lead element presented in perovskite solar cells could possibly leach out of the solar panel onto the rooftops or the soil below, even though the cells are encapsulated.

Increasingly broad types of lead-free compositions are being investigated to obtain new ones as the alternative elements, e.g. Sn, Ge, Cu etc. Amongst, the Sn was demonstrated to be capable of replacing/partly substituting for lead in perovskite solar cells^11^,^12^,^13^, although the organic Sn-based compounds were demonstrated to pass easily from the respiratory system to the bloodstream due to their solubility in fat^14^,^15^. Methylammonium tin halide perovskites (CH_3_NH_3_SnI_3−x_Br_x_) have been used as lead-free light harvesters for solar cell applications for the first time by Mercouri G. Kanatzidis *et al*., which enabled the realization of lead-free solar cells and lead to a promising initial PCE of 5.73%^16^. Meanwhile, they demonstrated that alloyed perovskite solid solutions of methylammonium tin iodide and its lead analogue (CH_3_NH_3_Pb_1−x_Sn_x_I_3_) are implemented as novel panchromatic light absorbing material in solar cell, which allowed extending the absorbance to 1050 nm^11^. Almost the same time, Henry Snaith and co-authors presented the CH_3_NH_3_SnI_3_ perovskite solar cell that processed on a mesoporous TiO_2_ scaffold, reaching efficiencies of over 6%^17^. Later on, Feng Hao *et al*. reported a solvent-mediated crystallization process to obtain a pinhole-free, highly uniform CH_3_NH_3_SnI_3_ thin film, in which the dimethyl sulfoxide (DMSO) solution was employed to form the transitional SnI_2_∙3DMSO intermediate phase^18^. Very recently, Seok *et al*. used the homogeneous dispersion of SnF_2_ through the formation of the SnF_2_-pyrazine complex to fabricate formamidinium tin iodide (FASnI_3_) PSCs with PCE of 4.8%^19^. Despite the fabrication was carried out in the nitrogen atmosphere in a glove box, the Sn^2+^ was easily oxidized into Sn^4+^ due to its instability. Therefore, the highest PCE of completely lead-free perovskite solar cells was only 6%, even for the Sn-involved perovskite solar cell, its maximum PCE still remained around 7%, which was not comparable to the lead-based perovskite solar cells that rose up to 22.1% recently. In this scenario, it is enlightening to realize competitive photovoltaic properties using lead-free organic–inorganic compounds toward commercial applications of perovskite solar cells.

In this communication, we used DMSO as the solvent mediation to form the PbI_2_/(SnI_2_)∙(DMSO)_x_ complexes, which gave rise to the formation of satisfied CH_3_NH_3_Pb_(1−x)_Sn_x_I_3_ (0 ≤ x ≤ 1) thin films. The tunable bandgap by different x values allowed realizing an efficient Sn-based inverted perovskite solar cell with a maximum PCE of 14.12%.

## Results

### Preparations and characterizations of CH_3_NH_3_Pb_(1−x)_Sn_x_I_3_ (0 ≤ x ≤ 1) perovskite thin films

Herein, the CH_3_NH_3_Pb_(1−x)_Sn_x_I_3_ (0 ≤ x ≤ 1) perovskite thin films were prepared by a two-step solution-processed technique that was able to control the crystallization rate of CH_3_NH_3_SnI_3_, which solved the problem of uncontrollable crystal growth due to its habitual nature of easier crystallization even at room temperature^17^. In our previous study, we employed DMSO to complex PbI_2_ in the case of pure CH_3_NH_3_PbI_3_^20^, by which, the obtained PbI_2_∙(DMSO)_x_ (0 ≤ x ≤ 1.86) complexes were proved to be capable of retarding the crystallization of PbI_2_. Here, the DMSO was used to complex the PbI_2_ and SnI_2_ separately via solvent engineering. We carefully studied the influence of precursor concentration of PbI_2_∙(DMSO)_x_ and SnI_2_∙(DMSO)_x_ on the thin films and the corresponding solar cells. The Sn precursor solution varied from 0 to 80 μL with a total volume (80 μL) of Sn and Pb as mentioned in the experimental section. The corresponding values of x in CH_3_NH_3_Pb_(1−x)_Sn_x_I_3_ were in the range from 0 to 1. It should be noted that the insitu complex thin films were amorphous and the corresponding XRD peaks were not able to be detected prior to post-annealing treatment. As shown in Supplementary Fig. 1 and Supplementary Fig. 2a, in the case of x = 0, the PbI_2_∙(DMSO)_x_ complex was formed as expected, which was consistent with our previous report^20^. While the Sn was introduced, the peaks that assigned to be SnI_2_∙(DMSO)_x_ complex were also observed (Supplementary Fig. 1). To further clarify the presence of the intermediates, we carried out the FTIR characterizations of the as-prepared intermediate layers (Supplementary Fig. 2), which were deposited from DMSO/dimethylformamide (DMF) solution before and after annealing. The FTIR spectra show the characteristic C−S and C−O stretching vibrations from the Sn^2+^- and/or Pb^2+^- coordinated DMSO solvent at 960 and 1012 cm^−1^, and C = O stretching vibrations from the Sn^2+^- and/or Pb^2+^- coordinated DMF solvent at 1389 and 1688 cm^−1^. After heated at 60 °C for 10 min, the stretching vibrations of C = O were disappeared while the C−S and C−O stretching vibrations were still exist. Combined with the XRD results (Supplementary Fig. 1), we can come to a conclusion that SnI_2_(DMSO)_x_ and/or PbI_2_(DMSO)_x_ were formed at relatively low temperatures, respectively^18^. Upon annealing at 140 °C, we found that the characteristic modes of the DMSO molecule disappeared, thus confirming the complete removal of the intermediate compound. Additionally, we found that the grain size of Sn-based complex had a significant enhancement in comparison to the one of PbI_2_-based complex, which was associated with the properties of easy crystallization and fast growth rate for SnI_2_ (Supplementary Fig. 3a,b). We assumed that the SnI_2_ dominated the complexed process in the copresence of SnI_2_ and PbI_2_ that dissolved in DMSO and DMF precursor solution. There was no any complex detected once the solvent without DMSO in the case x = 1 (Supplementary Fig. 1 and Fig. 3d). The highly crystallized SnI_2_ single crystal film with lower coverage prohibited the further process to fabricate solar cells.

**Figure 1:**
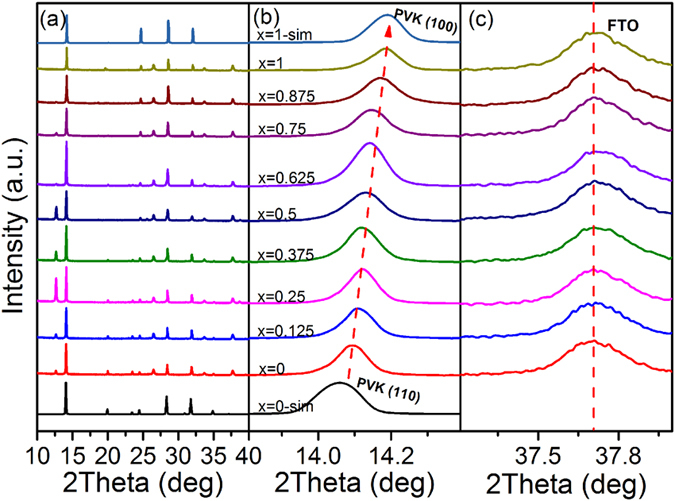
(**a**) XRD patterns of as-prepared CH_3_NH_3_Pb_(1−x)_Sn_x_I_3_ (0 ≤ x ≤ 1) thin films with different Sn concentrations; (**b**) The detailed transition of the peak varied from (110) to (100) with different Sn concentrations; (**c**) The corresponding FTO peaks.

As expected, the copresence of PbI_2_∙(DMSO)_x_ and SnI_2_∙(DMSO)_x_ complexes were demonstrated to slow down the crystallization of CH_3_NH_3_Pb_(1−x)_Sn_x_I_3_ (0 ≤ x ≤ 1) perovskite thin films. Figure 1 displays the XRD evolution of prepared CH_3_NH_3_Pb_(1−x)_Sn_x_I_3_ (0 ≤ x ≤ 1) thin films while the involved volume of Sn precursor solution varied from x = 0 to x = 1. Prior to XRD characterizations, we simulated the XRD patterns of x = 0 and x = 1, which represented the CH_3_NH_3_PbI_3_ and CH_3_NH_3_SnI_3_, respectively. The simulated results revealed that the perovskite phase altered from tetragonal phase (x = 0, CH_3_NH_3_PbI_3_, P4 mm (α-phase)) to cubic phase (x = 1, CH_3_NH_3_SnI_3_, I4 cm (β-phase)). As shown in Fig. 1, the (110) peak of perovskite gradually moved from 14.1° to 14.2° corresponding to (100) plane in cubic phase while the Sn introduction amount improved from x = 0 to x = 1, whereas the FTO peaks remained stable. Additionally, the two peaks located at 23.5° and 24.5° were respectively assigned to be (211) and (202) in the case of CH_3_NH_3_PbI_3_. With the x value increasing, these two peaks tent to merge to be a single peak corresponding to the (113) plane in the P4 mm space group (CH_3_NH_3_SnI_3_). Such evolution suggested a distortion of the relative positions of the octahedra along the 4-fold crystallographic axis. It turns out that the involved Sn induced the alteration of the perovskite phase, which is consistent with the previous report^11^.

Meanwhile, the superfluous PbI_2_/SnI_2_ appeared when the Sn involved inside the perovskite thin films as shown in Fig. 1a. It can also be certified by the morphology of perovskite thin film as shown in SEM images (Supplementary Fig. 4). As previously reported^21^,^22^,^23^, the sporadic formation of PbI_2_/SnI_2_ species in the grain boundaries might give rise to a successful passivation that forms energy barriers to prevent excitons from the surface defects and/or traps states.

**Figure 2:**
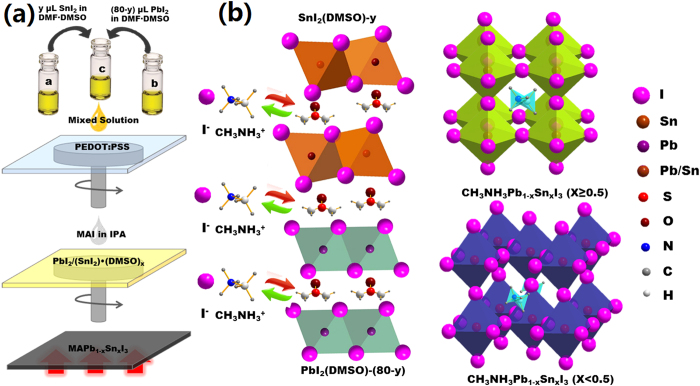
(**a**) Schematic of prepared process of CH_3_NH_3_Pb_(1−x)_Sn_x_I_3_ (0 ≤ x ≤ 1) thin films; (**b**) Schematic of formation mechanism in CH_3_NH_3_Pb_(1−x)_Sn_x_I_3_ (0 ≤ x ≤ 1) thin films and the corresponding crystal structure.

We then proposed the potential mechanism in the kinetic process of perovskite crystal growth in the presence of DMSO as shown in Fig. 2. Here, the PbI_2_/SnI_2_ can be intercalated by DMSO solvent mediation, which gives rise to the formation of PbI_2_/(SnI_2_)∙(DMSO)_x_ complexes (Fig. 2a). During the complexing process, the DMSO solvent is advantageous to the formation of amorphous mirror-like film as shown in Supplementary Fig. 3. Meanwhile, there might be a competed relationship while the PbI_2_/SnI_2_ complexes with DMSO. The SnI_2_ is supposed to govern the complexing process owing to its higher activation energy. Afterward, the presence of CH_3_NH_3_I (MAI) will exchange DMSO due to its higher affinity ability toward PbI_2_/SnI_2_ in comparison to DMSO, which allows the formation of CH_3_NH_3_Pb_(1−x)_Sn_x_I_3_ (0 ≤ x ≤ 1) thin films with highly dense and full coverage. As discussed above, the phase of perovskite thin film strongly depends on the x value. When x < 0.5, the structure is assigned to be tetragonal phase, whereas it is cubic phase once x ≥ 0.5 (Fig. 2b).

**Figure 3:**
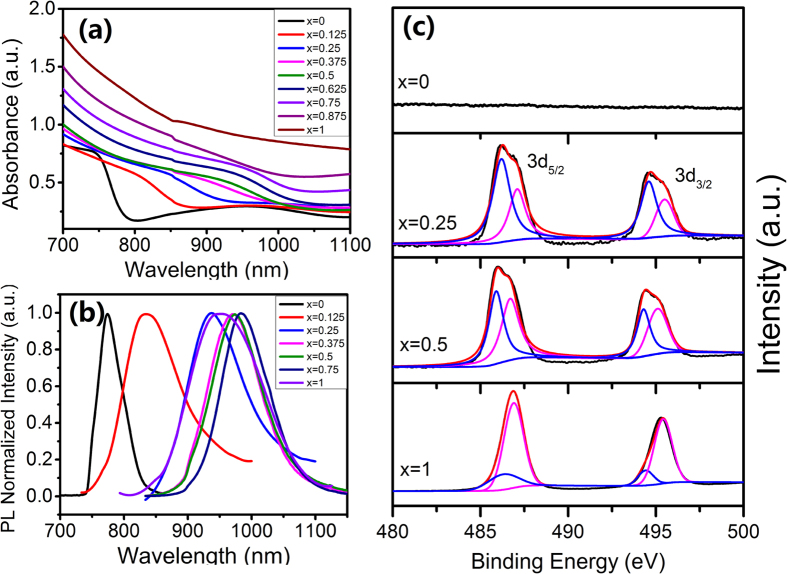
(**a**) UV-vis absorption spectra, (**b**) PL spectra and (**c**) XPS spectra of Sn3d_3/2_ and Sn3d_5/2_ of CH_3_NH_3_Pb_(1−x)_Sn_x_I_3_ (0 ≤ x ≤ 1) thin films with different Sn concentrations.

Figure 3a exhibits the UV-vis absorbance of series CH_3_NH_3_Pb_(1−x)_Sn_x_I_3_ (0 ≤ x ≤ 1) thin films. With the Sn content increased, the optical absorbance band-edge of thin film clearly moved toward infrared region in consideration of the narrowed bandgap of CH_3_NH_3_SnI_3_. Figure 3b displays the PL spectra of series CH_3_NH_3_Pb_(1−x)_Sn_x_I_3_ (0 ≤ x ≤ 1) thin films. In the case of x = 0, the PL spectrum was located at 775 nm, which was assigned to the recombination of electron-hole pair for the typical CH_3_NH_3_PbI_3_ thin film^24^,^25^. Again, the intermediate perovskite thin films exhibited an infrared shift with Sn content increasing. The corresponding PL spectrum was consequently fixed at 985 nm. In the case of x = 1, i.e. the case of pure CH_3_NH_3_SnI_3_ thin film, unexpectedly, the PL spectrum shift to shorter wavelength (955 nm) in comparison to the case of x = 0.75. Similar phenomenon was also observed in the previous report^11^, which was associated with the tunable bandgap for the intermediate-alloyed compounds of CH_3_NH_3_Pb_(1−x)_Sn_x_I_3_ (0 ≤ x ≤ 1).

It was demonstrated that the Sn^2+^ was easily oxidized into Sn^4+^, which likely cause the instability of Sn-based perovskite solar cells^19^. To study the stability of as-prepared CH_3_NH_3_Pb_(1−x)_Sn_x_I_3_ (0 ≤ x ≤ 1) thin films, we carried out its XPS characterizations (Fig. 3c). As expected, we did not detect any signal from the Sn element in the sample x = 0. Once the Sn involved, the peak that assigned to Sn element appeared, which was fitted into two main peaks, the corresponding peaks of 485.8 eV and 486.8 eV were associated with the Sn^2+^ and Sn^4+^, respectively. There was a tendency that the Sn^2+^ decreased, whereas the Sn^4+^ increased while enhancing the Sn content in the perovskite thin films. It appeared that the content of Pb is able to stabilize Sn in its 2+ state somehow. Likewise, the O peaks located at 530.5 eV that were fitted and assigned to SnO_2_, which tent to appear and strengthen with the Sn introduction amount increasing (Supplementary Fig. 5). This variation further certified that the CH_3_NH_3_Pb_(1−x)_Sn_x_I_3_ (0 ≤ x ≤ 1) thin films preferred to be oxidized while the Pb^2+^ content decreased as we mentioned previously. As expected, the peak intensity of Sn element gradually enhanced, whereas the one of Pb displayed a decreased trend with the Sn introduction amount increasing. Besides, the peak positions of Pb and I elements had a small shift while varying the x value, such fluctuation implies that the coordination environment of Pb and/or I was altered when Sn^2+^ was introduced inside the crystal lattice.

**Figure 4:**
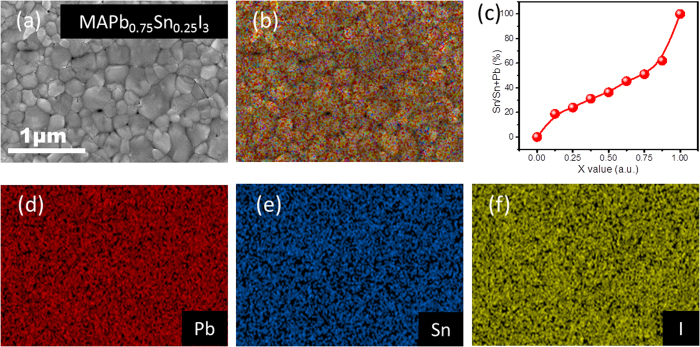
(**a**) Representative SEM images; (**b**,**d**,**e** and **f**) the corresponding EDS elemental mapping of Pb, Sn and I in CH_3_NH_3_Pb_0.75_Sn_0.25_I_3_, the scale bar is 1 μm. (**c**) the calculated ratio of Sn:(Sn+Pb) with different Sn introduction amount.

Figure 4 shows the representative SEM images and the corresponding EDS element mapping of Pb, Sn and I in the CH_3_NH_3_Pb_0.75_Sn_0.25_I_3_ film. Apparently, the Sn, Pb and I were homogeneously distributed throughout the film without any obvious phase separation. We also quantified the atomic ratio of Sn over Sn+Pb as the function of Sn introduction amount by EDS analysis as shown in Fig. 4c. We note that significantly less Sn was presented in the final perovskite than added to the precursor in the intermediary concentrations. The loss of the Sn in the final perovskite films was likely associated with the lower solubility of Sn^2+^ in isopropanol. Particularly, in such two-step process, while the CH_3_NH_3_I precursor solution in isopropanol was dropped on the SnI_2_/PbI_2_ film, a little amount of SnI_2_ was dissolved and thrown off with the spin coating process. Supplementary Fig. 4 clearly demonstrates the morphology evolution of CH_3_NH_3_Pb_(1−x)_Sn_x_I_3_ thin films from x = 0 to x = 1. With the Sn content increasing, the grain sizes of perovskite tent to be enlarged. However, the roughness and coverage of the film suddenly become very poor in the case of x = 1, which further certifies that the presence of lead is able to stabilize the Sn perovskite thin films as mentioned above. Likewise, considering that the growth rate of SnI_2_-based complex was faster than that of PbI_2_-based complex, the cubic phase of CH_3_NH_3_Pb_(1−x)_Sn_x_I_3_ (x ≥ 0.5) thin films were easier to obtain. In such two-step process, we can immediately obtain the cubic Sn-rich perovskite film once spin coating MAI precursor solution at room temperature, which usually gave rise to the film with bigger crystal size but a lower coverage (Supplementary Fig. 4). In this regard, the current two-step procedure was favorable to prepare the Pb-rich and Sn-poor perovskite films. One-step solution-process with the same precursor solution might be more suitable to prepare Pb-free perovskite films.

### Photovoltaic performances of CH_3_NH_3_Pb_(1−x)_Sn_x_I_3_ (0 ≤ x ≤ 1) perovskite solar cells

**Figure 5:**
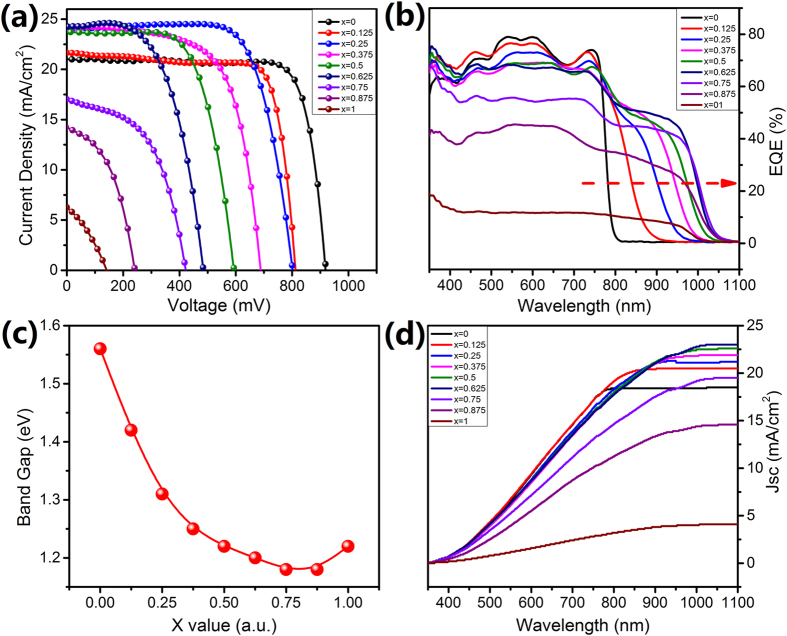
(**a**) I–V curves of CH_3_NH_3_Pb_(1−x)_Sn_x_I_3_ (0 ≤ x ≤ 1) solar cells with different Sn concentrations; (**b**) the corresponding EQE spectra of the cells; (**c**) the calculated bandgap of CH_3_NH_3_Pb_(1−x)_Sn_x_I_3_ (0 ≤ x ≤ 1) thin films with different Sn concentrations; (**d**) the integrated J_sc_ based on the EQE results.

Perovskite solar cells with an inverted structure of FTO/PEDOT:PSS/Perovskite/PCBM/BCP/Ag were then fabricated by the series CH_3_NH_3_Pb_(1−x)_Sn_x_I_3_ (0 ≤ x ≤ 1) thin films (Supplementary Fig. 6). The use of PEDOT:PSS as hole transport material (HTM) can effectively avoid deteriorating the cells by the commonly used HTM including spiro-OMeTAD, lithiumbis(trifluoromethylsulfonyl) imide salt, and 4-tert-butylpyridine. Figure 5a gives the I–V curves of corresponding cells. The CH_3_NH_3_PbI_3_ solar cell shows a decent fill factor (FF) of 80.9%, short-circuit photocurrent density (J_sc_) of 20.98 mAcm^−2^, an open-circuit voltage (V_oc_) of 0.918 V, yielding a PCE of 15.58% under AM1.5G solar illumination. While the Sn species were introduced inside the perovskite thin film, the V_oc_ of corresponding cell decreased, whereas the J_sc_ had a notable enhancement in virtue of the extending absorbed edge. The decreased V_oc_ was ascribed to the lower conduction band edge with decreasing Pb content^26^. After carefully optimized the technic parameters, the maximum PCE of CH_3_NH_3_Pb_0.75_Sn_0.25_I_3_ perovskite solar cells can rise up to 14.12%, which, to our knowledge, is the highest reported value so far. In the present study, x = 0.25 appears to be the optimum condition by means of banlancing the tradeoff between Pb-content and photovoltaic performances. We then characterized the I–V curves of CH_3_NH_3_PbI_3_ and CH_3_NH_3_Pb_0.75_Sn_0.25_I_3_ by using 55 cells and gave the statistics on photovoltaic results, forward and backward scan as shown in Table 1. The results show that both CH_3_NH_3_PbI_3_ and CH_3_NH_3_Pb_0.75_Sn_0.25_I_3_ cells have relatively lower hysteresis effects. Likewise, we futher checked the stability under illumination of the CH_3_NH_3_PbI_3_ and CH_3_NH_3_Pb_0.75_Sn_0.25_I_3_ cells, which was shown in Supplementary Fig. 7. In comparision to pure Pb-based device, more or less Sn-involved inside the perovskite thin films would lead to inferior stability of the corresponding cells, which was consistent with the previous report^18^. The oxidation of Sn^2+^ in air might be the main cause that quenched the photovoltaic poerfomances of the Sn-based solar cells. Careful encapsulation technology is under study and would resolve the instability of Sn-based solar cell. It is worthy of noting that the PCE of cells with different amount of Sn species here overall outperformed the ones with same Sn content in previous report^11^. As shown in Fig. 5b, the external quantum efficiency (EQE) of series CH_3_NH_3_Pb_(1−x)_Sn_x_I_3_ (0 ≤ x ≤ 1) solar cells were demonstrated to cover the whole visible spectrum and realize a broad absorption minimum over 50% from 350 to 950 nm accompanied by a noteworthy absorption onset up to 1050 nm. It should be mentioned that the CH_3_NH_3_SnI_3_ solar cell had an ultraviolet-shift in comparison to the case of CH_3_NH_3_Pb_0.25_Sn_0.75_I_3_, which was consistent with the PL characterizations. We calculated the band gap of CH_3_NH_3_Pb_(1−x)_Sn_x_I_3_ (0 ≤ x ≤ 1) thin films based on the EQE (Fig. 5c). It clearly pointed out that we can easily tune the bandgap of perovskite thin film between 1.18 and 1.56 eV. The intermediate compounds with x = 0.75 exhibited the smallest bandgap of 1.18 eV. Meanwhile, we integrated the J_sc_ that estimated from EQE (Fig. 5d), which were in consistent with the measured J_sc_ as shown in Fig. 5a.

## Discussion

The pure Sn-based perovskite thin film still suffered from the poor perovskite film quality and low coverage, which gave rise to a poor photovoltaic performance. In the present system, we assumed that the pure SnI_2_ was not able to effectively complex the DMSO to form the intermediate compound in view of its higher activation energy toward crystallization, thus enabling the SnI_2_ to crystallize quickly just as the case without any solvent mediation. Moreover, the presence of Sn^4+^ in CH_3_NH_3_SnI_3_ thin film might also contribute to the quenched J_sc_. More studies on the stability of CH_3_NH_3_SnI_3_ solar cells, e.g. solvent engineering, professional encapsulation are underway.

In summary, we employed DMSO to intercalate inside the lattice structure of PbI_2_/SnI_2_, which allowed the formation of intermediate complexes of PbI_2_/(SnI_2_)∙(DMSO)_x_, by which, the CH_3_NH_3_Pb_(1−x)_Sn_x_I_3_ (0 ≤ x ≤ 1) thin films with full coverage and decent grain size can be obtained. The tunable bandgap and high quality of Sn-introduced perovskite thin films facilitated the realization of solar cells with maximum PCE of 14.12%. We believe that our study can shed lights on the realization of highly efficient Pb-free perovskite solar cells.

## Methods

### Film preparation and device fabrication

The fluorine doped tin oxide (FTO)-coated glass (8 Ω/cm^2^, Nippon) was cleaned with deionized water, acetone and alcohol in an ultrasonic washing unit by turn. After dried under N_2_ atmosphere, the glass was treated under oxygen plasma for 10 min. PEDOT:PSS (Heraeus-Clevios P 4083, Xi’an p-OLED) was spun on the as-treated substrate at a speed of 3,500 rounds per minute (r.p.m.). The film was then annealed at 140 °C for 10 min. 170 mg of PbI_2_ (Alfa Aesar, 99.9985%) and 138 mg SnI_2_ (Aldrich, 99.99%) were dissolved in the mixed solvent containing 200 μL of DMF (Aldrich, 99.9%) and 40 μL of DMSO (Aldrich, 99.9%), respectively. After heated at 60 °C for 2 h, a certain amount of prepared PbI_2_ and SnI_2_ solutions were mixed to form the precursor solution. It’s worthy of noting that the PbI_2_ solution needed to be filtered prior to mixing. The mixed precursor solution was spun on the PEDOT:PSS layer at 5000 (r.p.m.) for 30 s. After natural evaporation for 10 min to form the PbI_2_(SnI_2_)·(DMSO)_x_ complexes, the MAI solution with the concentration of 60 mg/ml was spun on the substrate at 4000 (r.p.m.) for 30 s. Afterward, the obtained thin films were annealed at 120 °C for 20 min. Exceptionally, the pure PbI_2_-based perovskite film was annealed at 140 °C for 20 min. The [6,6]-phenyl-C61-butyric acid methyl ester (PCBM) dissolved into dichlorobenzene with the concentration of 20 mg/ml was spun on the top of the as-prepared perovskite layers at 2000 (r.p.m.) for 30 s. Finally, 6 nm 2,9-dimethyl-4,7-diphenyl-1,10-Phenanthroline (BCP) and 120nm Ag electrode were sequentially deposited by thermal evaporation. Note that all of the film fabrications were processed in nitrogen-filled glovebox.

### Characterizations

The crystal structure was characterized by Bruker D8 Advance X-ray diffractometer (XRD) with CuKα radiation at 40 kV and 40 mA. Field-emission scanning electron microscopy (SEM) was used to characterize the morphology of the obtained thin film. Both top-down and cross-sectional views were obtained using a FEI NovaNanoSEM450. A double beam spectrophotometer (U-4100, Hitachi) equipped with an integrated sphere was used for the UV-vis transmission measurements in the range from 700 to 1100 nm. A Fourier transform infrared spectroscopy was used to collect the FT-IR spectral data in the 4000 cm^−1^–400 cm^−1^ range. The KBr pellet was used for the powdered samples of layer materials scraped from the substrate. X-ray photoelectron spectroscopy (XPS) was measured with a PHI 5300 ESCA Perkin-Elmer spectrometer. All spectra were shifted to account for sample charging using inorganic carbon at 284.80 eV as a reference. The photoluminescence (PL) was carried out under the excitation of a 532-nm continuous-wave laser. The PL signal is sent to a 0.5-m spectrometer through a 50× objective lens and then detected by a liquid nitrogen-cooled CCD detector array. All PL spectrums were normalized.

Current-voltage (J-V) characteristics of perovskite solar cells were measured using a semiconductor device analyzer (Keithley 2601B) and a SAN-EI solar simulator (XES-100S1) with an AM 1.5 G spectrum. The illumination power on the sample was adjusted to 1000 W m^−2^ using a certified reference solar cell (RS-ID-4). A black mask with an aperture (9 mm^2^) was placed on the top of the device to control the effective electrode area. Both forward and backward scans were performed and the scan speed was fixed at 0.15 V/s. The external quantum efficiency (EQE) of perovskite solar cell device was measured by using spectrum corresponding system (QTesT 1000ADX), with the monochromatic light wavelength ranging from 350 nm to 1100 nm. The monochromatic light beam is produced by a dual grating monochromatic in front of a halogen lamp. A Si reference solar cell with known EQE is used to determine the spectral response of perovskite solar cells.

## Additional Information

**How to cite this article**: Liu, C. *et al*. Highly Efficient Perovskite Solar Cells with Substantial Reduction of Lead Content. *Sci. Rep.* **6**, 35705; doi: 10.1038/srep35705 (2016).

## Supplementary Material

Supplementary Information

## Figures and Tables

**Table 1:** Statistics of device performance of CH_3_NH_3_PbI_3_ and CH_3_NH_3_Pb_0.75_Sn_0.25_I_3_ cells.

Samples		V_oc_/mV	J_sc_/mA∙cm^−2^	FF/%	PCE/%
CH_3_NH_3_PbI_3_ Average	Forward	944 ± 19.3	20.29 ± 1.2	71.11 ± 3.25	13.61 ± 0.68
	Reverse	941 ± 13.5	19.86 ± 1.05	71.02 ± 2.7	13.3 ± 1.07
CH_3_NH_3_PbI_3_ Champion	Forward	957.8	20.65	74.16	14.67
	Reverse	957.8	20.24	73.30	14.21
CH_3_NH_3_Pb_0.75_Sn_0.25_I_3_ Average	Forward	757 ± 22.7	22.82 ± 1.29	70.9 ± 2.6	12.25 ± 0.76
	Reverse	759 ± 21.3	22.35 ± 1.26	74.48 ± 3.1	12.63 ± 0.77
CH_3_NH_3_Pb_0.75_Sn_0.25_I_3_ Champion	Forward	745.4	23.87	74.74	13.3
	Reverse	745.4	23.8	78.56	13.93
