# Supplementary material for: Highly Efficient Perovskite Solar Cells with Substantial Reduction of Lead Content
Source: Sci Rep. 2016 Oct 18;6:35705. doi: 10.1038/srep35705 (PMC5067674; doi:10.1038/srep35705)
Supplement: Supplementary Information [file srep35705-s1.pdf]

# Highly Efficient Perovskite Solar Cells with Substantial Reduction of Lead Content

*Chong Liu[†], Jiandong Fan[†‡*], Hongliang Li[†], Cuiling Zhang[†], Yaohua Mai[†‡*]*

*[†]Institute of Photovoltaics, College of Physics Science and Technology, Hebei University, Baoding, 071002, China*

*[‡] Institute of New Energy Technology ,College of Information and Technology, Jinan University, Guangzhou, 510632, China*

*E-mail: (J. F.) jdfan@jnu.edu.cn;*

*E-mail: (Y. M.) yaohuamai@jnu.edu.cn.*

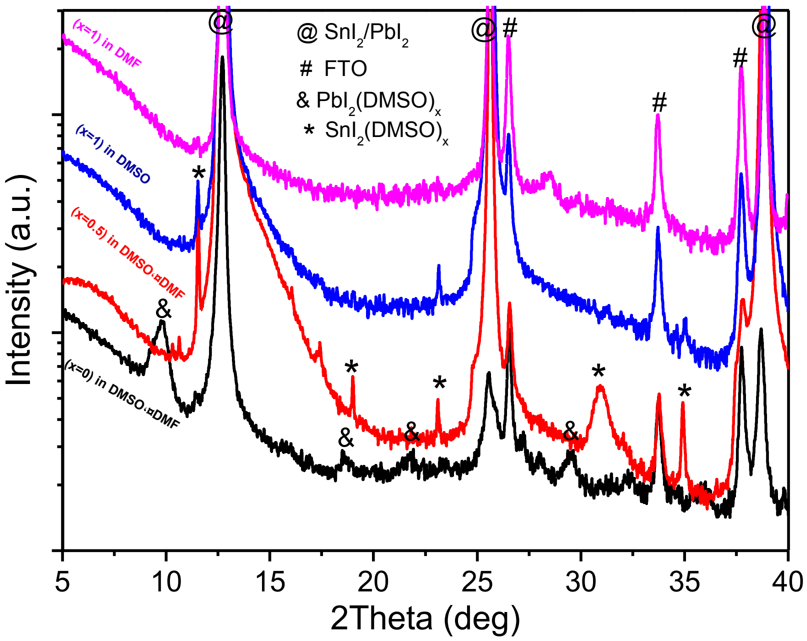


**Supplementary Figure 1.** XRD patterns of $PbI_2/(SnI_2)$ (DMSO)$_x$ complexes with different Sn concentrations after post annealing treatment at 90 ℃ for 10 min.

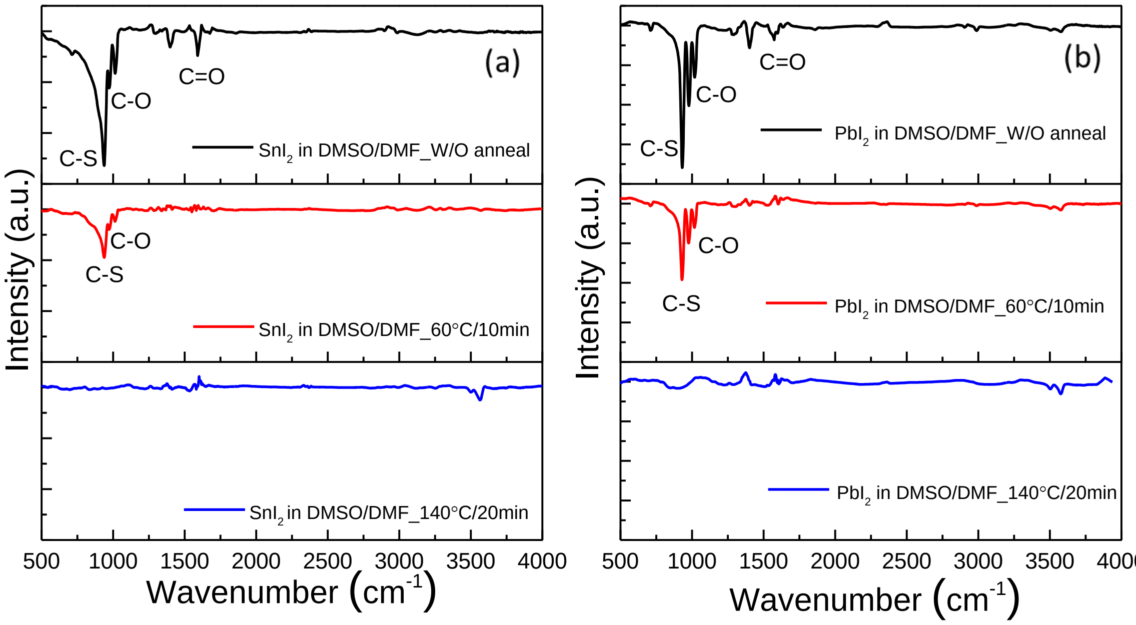


**Supplementary Figure 2.** FTIR spectra of the perovskite layer deposited from DMSO/DMF solution before and after annealing showing the characteristic C−S and C−O stretching vibrations from the $Sn^{2+}$- and $Pb^{2+}$- coordinated DMSO solvent at 960 and 1012 $cm^{-1}$, and C=O stretching vibrations from the $Sn^{2+}$- and/or $Pb^{2+}$- coordinated DMF solvent at 1389 and 1688 $cm^{-1}$. (a) $SnI_2$ (DMSO)x intermediates; (b) $PbI_2$ (DMSO)x intermediates.

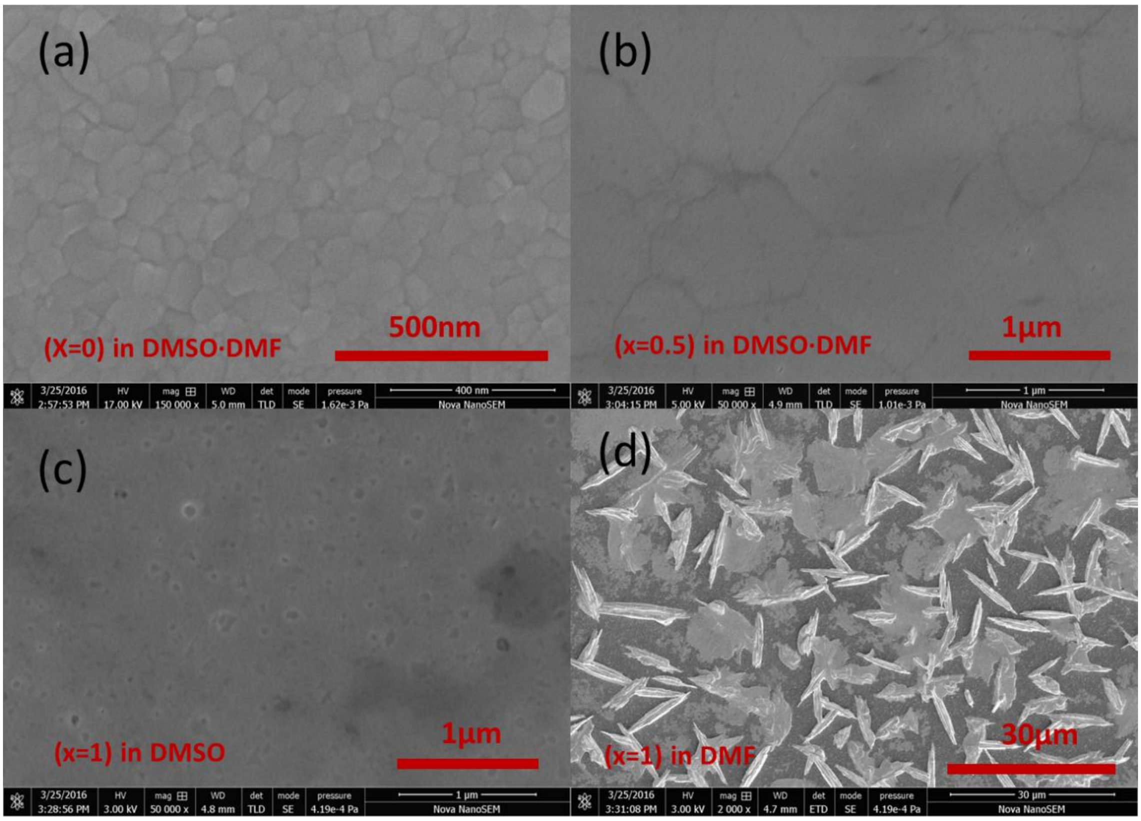


**Supplementary Figure 3.** SEM images of $PbI_2/(SnI_2)$ $(DMSO)_x$ complexes with different Sn concentrations.

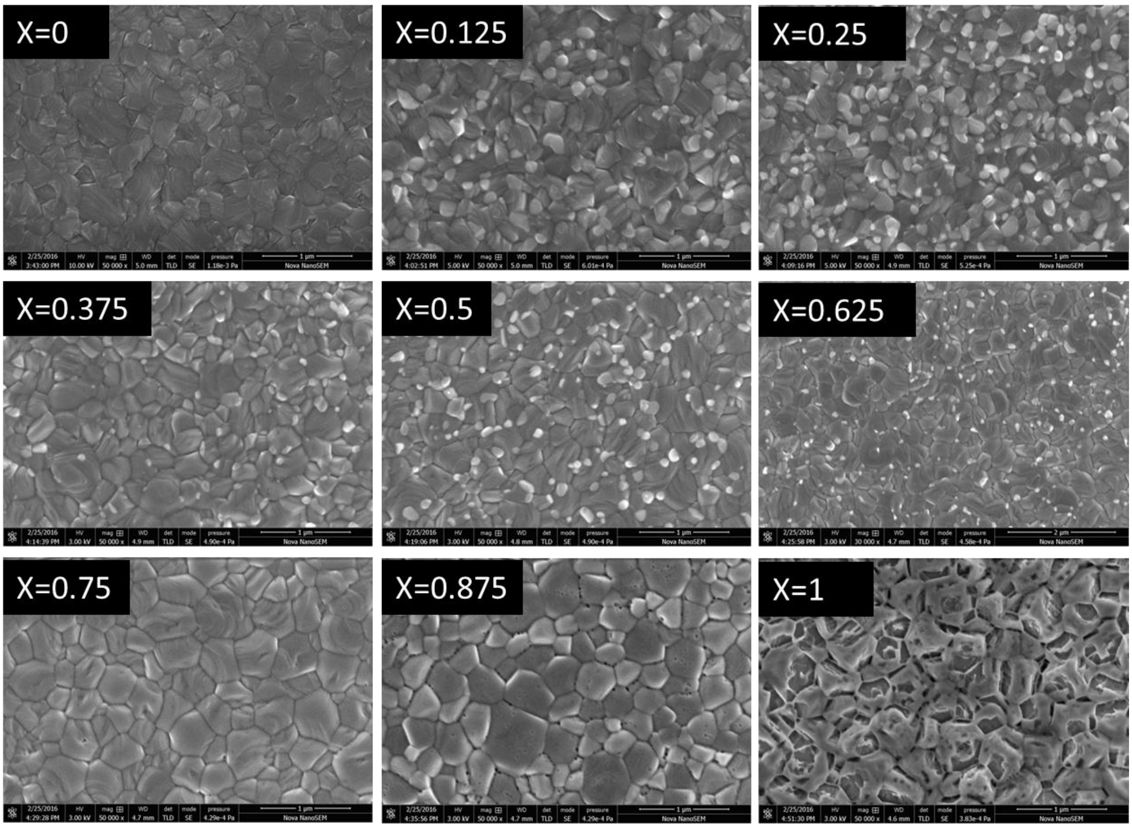


**Supplementary Figure 4.** SEM images of $CH_3NH_3Pb_{(1-x)}Sn_xI_3$ ($0 \leq x \leq 1$) thin films with different Sn concentrations.

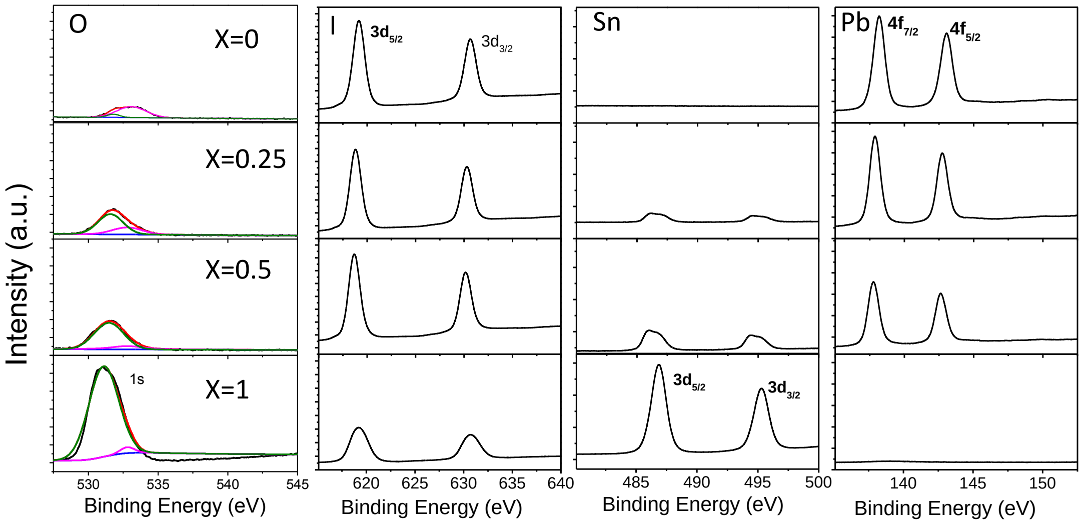


**Supplementary Figure 5.** XPS spectra of Pb, I, Sn and O in $CH_3NH_3Pb_{(1-x)}Sn_xI_3$ ($0 \leq x \leq 1$) thin films with different Sn concentrations.

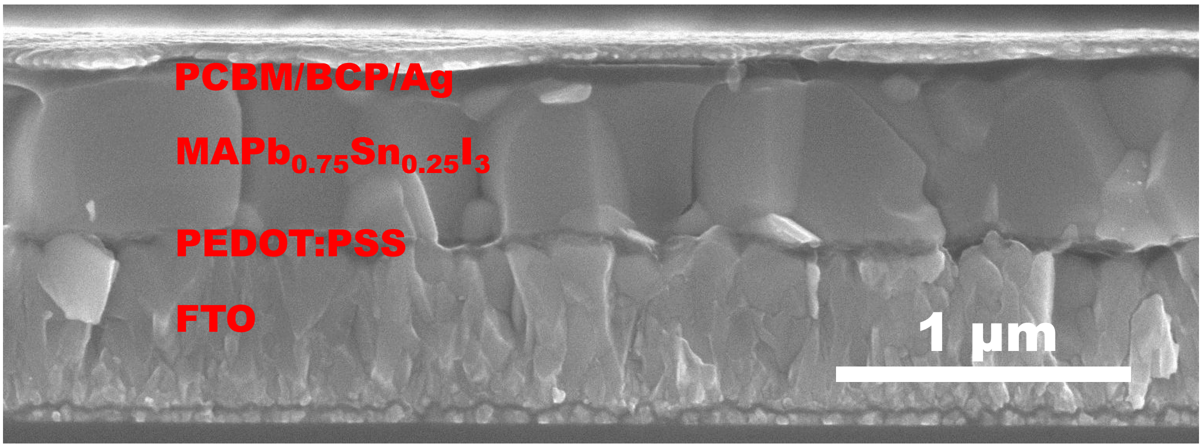


**Supplementary Figure 6.** The cross-sectional SEM image of $CH_3NH_3Pb_{0.75}Sn_{0.25}I_3$ solar cell with inverted structure.

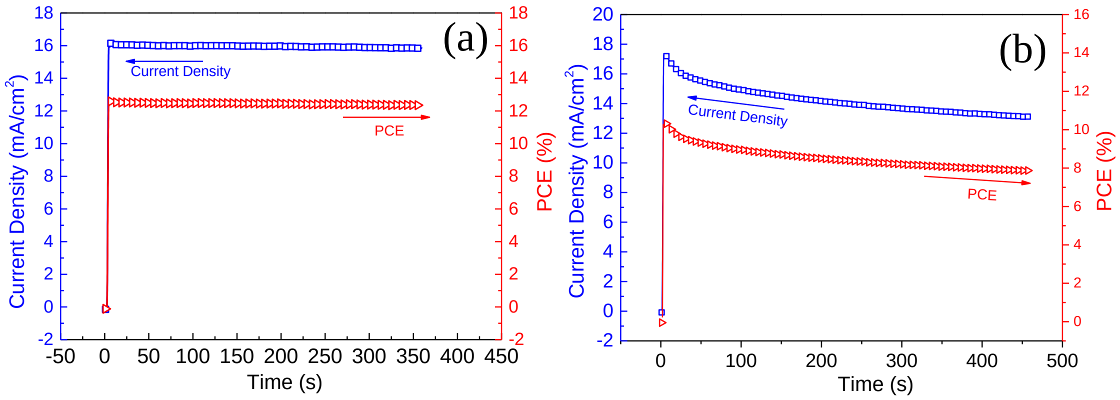


**Supplementary Figure 7.** Photocurrent density and power conversion efficiency as a function of time for the same cell held close to 0.79 V and 0.60 V forward bias, respectively. (a) $CH_3NH_3PbI_3$; (b)$CH_3NH_3Pb_{0.75}Sn_{0.25}I_3$
